# Curcumin Suppressed Activation of Dendritic Cells via JAK/STAT/SOCS Signal in Mice with Experimental Colitis

**DOI:** 10.3389/fphar.2016.00455

**Published:** 2016-11-25

**Authors:** Hai-Mei Zhao, Rong Xu, Xiao-Ying Huang, Shao-Min Cheng, Min-Fang Huang, Hai-Yang Yue, Xin Wang, Yong Zou, Ai-Ping Lu, Duan-Yong Liu

**Affiliations:** ^1^School of Basic Medical Sciences, Jiangxi University of Traditional Chinese MedicineNanchang, China; ^2^Department of Postgraduate, Jiangxi University of Traditional Chinese MedicineNanchang, China; ^3^Key Laboratory of Modern Preparation of TCM, Ministry of Education, Jiangxi University of Traditional Chinese MedicineNanchang, China; ^4^School of Chinese Medicine, Hong Kong Baptist UniversityKowloon Tong, China; ^5^Science and Technology College, Jiangxi University of Traditional Chinese MedicineNanchang, China

**Keywords:** curcumin, dendritic cell, experimental colitis, JAK/STAT/SOCS signal, costimulatory molecules

## Abstract

Dendritic cells (DCs) play a pivotal role as initiators in the pathogenesis of inflammatory bowel disease and are regulated by the JAK/STAT/SOCS signaling pathway. As a potent anti-inflammatory compound, curcumin represents a viable treatment alternative or adjunctive therapy in the management of chronic inflammatory bowel disease (IBD). The mechanism of curcumin treated IBD on DCs is not completely understood. In the present study, we explored the mechanism of curcumin treated experimental colitis by observing activation of DCs via JAK/STAT/SOCS signaling pathway in colitis mice. Experimental colitis was induced by 2, 4, 6-trinitrobenzene sulfonic acid. After 7 days treatment with curcumin, its therapeutic effect was verified by decreased colonic weight, histological scores, and remitting pathological injury. Meanwhile, the levels of major histocompatibility complex class II and DC costimulatory molecules (CD83, CD28, B7-DC, CD40, CD40 L, and TLR2) were inhibited and followed the up-regulated levels of IL-4, IL-10, and IFN-γ, and down-regulated GM-CSF, IL-12p70, IL-15, IL-23, and TGF-β1. A key finding was that the phosphorylation of the three members (JAK2, STAT3, and STAT6) of the JAK/STAT/SOCS signaling pathway was inhibited, and the three downstream proteins (SOCS1, SOCS3, and PIAS3) from this pathway were highly expressed. In conclusion, curcumin suppressed the activation of DCs by modulating the JAK/STAT/SOCS signaling pathway to restore immunologic balance to effectively treat experimental colitis.

## Introduction

Inflammatory bowel disease (IBD), including ulcerative colitis (UC) and Crohn’s disease (CD), is an idiopathic disease characterized by chronic, relapsing, non-specific inflammatory reactions in the bowel. Although the exact etiology of IBD is unknown, the accepted etiology is that dysregulated immune responses in the enteric mucosa lead to local inflammation (Bouma and Strober, 2003; Bamias and Cominelli, 2007; Iwakami et al., 2009). During the pathogenic process of the disease, DCs play the role of pivotal initiator in the abnormal immune response that relaxes mucosal vigilance against intestinal flora (Steinman, 2012; Al-Hassi et al., 2014).

As the most powerful antigen-presenting cell, intestinal DCs distribute throughout the non-lymphoid and lymphoid tissues including lamina propria (LP), Peyer’s patches (PPs), Mesenteric lymph nodes (MLN), and so on. (Rimoldi et al., 2005; Verstege et al., 2008). When DCs are in an immature state, they are unable to activate T cells due to a lack of essential T cell signals of activation (i.e., TLR, CD40, CD83, interleukin (IL)-12 p70, IL-23), further maintaining immune tolerance (Abe et al., 2007; Berndt et al., 2007).

Together with immunogenicity, intestinal DCs jointly maintain the dynamic balance in the gut between immunogenicity against invading pathogens and tolerance of the commensal microbiota. Disruption of the balance can weaken mucosal immunogenicity against the pathogenic antigen and destroy the protection from commensal intestinal bacteria (Al-Hassi et al., 2014).

Over-maturation or dysfunction in DCs can promote inflammatory cells (including primed T cells) to secrete excessive inflammatory mediators, which further compromise the balance between pro-inflammatory and anti-inflammatory responses, to destroy the intestinal mucosal barrier, and to induce IBD (Iliev et al., 2009; Peña et al., 2009; Baumgart et al., 2011).

The search for a suitable signaling pathway in the maturation and activation of DCs to treat IBD is ongoing. Heine et al., 2013 found that the Janus kinase (JAK)–inhibitor, ruxolitinib, reduced DC activation, and treated myelofibrosis by deregulating JAK-STAT (signal transducer and activator of transcription) signaling. JAK-STAT signaling pathway can selectively influence the maturation, activation, and function of DCs by the JAK protein family, downstream STAT proteins, and types I and II cytokine receptors associated with JAK (Schindler et al., 2007; Kim et al., 2015). However, there is little evidence that drugs can regulate DC maturation or activate JAK-STAT signaling to treat IBD.

Curcumin, a major component of turmeric, is a natural phenol and yellow pigment from the rhizomes of Curcuma longa (Family: Zingiberaceae; Huang et al., 2008; Kumar et al., 2012). Since curcumin was first shown to exhibit antibacterial activity in 1949 (Schraufstatter and Bernt, 1949), many studies have demonstrated that curcumin exhibits several pharmacological actions that include anti-infectious, anti-oxidant, anti-inflammatory, and anti-carcinogenic properties. Curcumin is a highly pleiotropic molecule and has been shown to modulate the biological activity of signaling pathways [such as JAK/STAT signaling, the mitogen-activated protein kinases (MAPK) pathway, and the ERK pathway] and multiple molecular targets (such as IL-2, IL-6, IL-8, TGF-β, TNF-α, ICAM-1, STAT1, STAT3, and MMP9; Gupta et al., 2013; Shanmugam et al., 2015). As a potent anti-inflammatory compound, curcumin represents a viable treatment alternative or adjunctive therapy in the management of chronic IBD (Epstein et al., 2010; Sareen et al., 2013).

The protective effects of curcumin treated IBD may be related to the inhibition of the nuclear factor κB (NF-κB) pathway, signal transduction and activation of transcription 3 (STAT3) proteins, p38 MAPK activity, and reduction of the pro-inflammatory Th1 cytokine response (Vecchi Brumatti et al., 2014; Shanmugam et al., 2015).

Interestingly, Zhang et al. (2016) found that curcumin attenuated inflammatory damages induced by 2, 4, 6-trinitrobenzene sulfonic acid (TNBS), which is realized by enhancing suppressors of cytokine signaling (SOCS)-1 expression and inhibiting JAK/STAT pathways. But they mainly observed that curcumin efficiently suppressed the TNBS-induced apoptosis, pro-inflammatory cytokines secretion and M1/M2 ratio, enhanced anti-inflammatory cytokines expression (Zhang et al., 2016). However, they do not indicate effect of curcumin on DCs from TNBS-induced colitis. It is known that DCs play important role in the pathogenic course of IBD. Moreover, curcumin prevents DCs from inducing CD4^+^T cell proliferation by inhibiting maturation markers, cytokine and chemokine expression, and by reducing both migration and endocytosis (Shirley et al., 2008). Although these results indicate that curcumin may regulate DCs to treat many chronic diseases, the effects and signaling targets of curcumin modulated DCs to treat IBD are unclear. Thus, the scientific hypothesis is that curcumin can treat IBD by regulating DCs mature or activation related to the JAK/STAT/SOCS signaling pathway. Therefore, we explored the actions of curcumin by observing expressions of costimulatory molecules, balance of T helper (Th) 1 and 2, the DCs levels and proteins of JAK/STAT/SOCS signaling pathway in mice with colitis induced by TNBS.

## Materials and Methods

### Mice

Male C57BL/6 mice were obtained from the Animal Center of Peking University Health Science Center (Animal certificate number SCXK 2012-0001). All mice were 9–12 weeks in age, caged under controlled conditions [light (12-h light/dark cycle), humidity (50% ± 5%), and temperature (23 ± 2°C)], and provided a standard diet and water *ad libitum* for the duration of the experiment. Animals were acclimatized for 3 days prior to starting the study. All animals were handled in accordance with the guidelines on animal welfare according to the Institutional Animal Care and Use Committee (IACUC) of Jiangxi University of Traditional Chinese Medicine (JXUTCM). The protocol (Permit number: JZ2015-016) of the present study was approved by IACUC.

Thirty-two mice were randomly assigned to four groups with eight mice in each group: the Normal group (Normal), the TNBS group (TNBS), the TNBS + Curcumin group, and the TNBS + Mesalazine group.

### Drugs

Curcumin (batch number: GR-133-140421, purity >95% by HPLC) was obtained from GANGRUN Biotechnology (Nanjing, China), and TNBS (batch number: p2297) was purchased from Sigma (St. Louis, MO, USA). Mesalazine (batch number: 130407) was purchased from Sunflower Pharma (Jiamusi, China).

### Induction of Experimental Colitis

As previously described (Fina et al., 2008; Bai et al., 2010; Schmidt et al., 2010; Huang et al., 2014; Sałaga et al., 2014), experimental colitis was induced with TNBS. Briefly, to have an unobstructed enema, a deprivation of food for 24 h with free access to a 5% glucose solution and a randomization of mice had been performed. C57BL/6 mice were lightly anesthetized with pentobarbital sodium (40 mg/kg) by intraperitoneal injection, and 100 mg/kg TNBS (dissolved in 0.3 mL 50% ethanol) was infused into the colon approximately 4 cm from the anus. To assure TNBS/ethanol solution distributed into the entire colon, the mouse was maintained in a head-down position for 5 min. Control animals in the normal group received the same volume of vehicle alone (0.3 mL of 50% ethanol).

### Pharmacological Treatments

Before administration, curcumin was dissolved in 5% dimethyl sulfoxide (DMSO) in physiological saline, which was used as the vehicle. Twenty-four hours after TNBS infusion, the mice in the TNBS + Curcumin and TNBS + Mesalazine groups were, respectively, administered curcumin (100 mg/kg; purity >95% by HPLC, batch number: GR-133-140421, GANGRUN Biotechnology, Nanjing, China) or Mesalazine (300 mg/kg; batch number: 130407, Sunflower Pharma, Jiamusi, China) by oral gavage for 7 days. In the two other groups, all animals were administered the same volume of 5% DMSO in physiological saline for 7 days.

### Evaluation of Colonic Damage

On the eight day, all mice were sacrificed after having been anesthetized with pentobarbital sodium (40 mg/kg) by intraperitoneal injection. The colon was removed rapidly and its length was measured, opened longitudinally, rinsed with phosphate buffered saline (PBS), assessed immediately for weight (*n* = 8 for each group), and the weight index of colon was computed (*n* = 8; colonic weight/body weight × 100%). Then, segments of the colon were fixed in 4% polyformaldehyde solution for at least 7 days. Subsequently, colon tissues were dehydrated, embedded in paraffin, sectioned at 5 μm, and mounted onto slides. These sections were stained with hematoxylin and eosin (*n* = 8).

A histological damage score (*n* = 8) was determined according to the criteria described by Nicole and Alexander et al (Schmidt et al., 2010). The histological score included inflammatory cell infiltration and tissue damage. Scores for infiltration were as follows: 0: no infiltration; 1: an increased number of inflammatory cells in the LP; 2: inflammatory cells extending into the submucosa; and 3: transmural inflammatory cell infiltration. The scores of tissue damage were as follows: 0: no mucosal damage; 1: discrete epithelial lesions; 2: erosions or focal ulcerations; and 3: severe mucosal damage with extensive ulceration extending into the bowel wall.

### Isolation of Lymphocyte from PPs

PPs (*n* = 8) were separated and collected from the small intestine. PPs were triturated in a 3% fetal calf serum (FCS)/PBS solution on ice and filtrated via 300 section stainless steel cell cribble. Lymphocytes from PPs were centrifuged at 1,600 × *g* at 4°C for 5 min and suspended into 1 × 10^6^∼10^7^/mL in 3% FCS/PBS solution.

### Assay of Costimulatory Molecules of DCs by Flow Cytometry (FCM)

The obtained lymphocytes were incubated with fluorescence conjugated monoclonal antibodies in staining buffer. Eight-color FCM analysis (*n* = 8) was performed on a FACS calibur (Becton-Dickinson, Mountain View, CA, USA) device. The frequency of positive cells was analyzed using the program Cell Quest in two regions. The lymphocyte region was determined using granularity (SSC) and size (FSC) plot. DCs were identified as an MHC^+^lineage^+^ (CD40^+^, CD40L^+^, CD28^+^, CD273^+^, CD83^+^, and CD86^+^) population, and within this group, the CD11c^+^ population was assessed. The following mAbs were used: APC/Cy7 anti-mouse CD11c (1: 300), PE Anti-Mouse CD40 (1: 300), APC Anti-Mouse CD154 (CD40 Ligand) (1: 300), PerCP-Cy^TM^5.5 Rat Anti-Mouse I-A/I-E (MHC-II) (1: 100), PerCP-Cyanine5.5Anti-Mouse CD28 (1: 300), FITC Anti-Mouse CD273 (B7-DC) (1: 200), FITC Anti-Mouse CD282 (TLR2) (1: 100), Alexa Fluor^®^ 700 Rat Anti-Mouse CD86 (1: 300), and APC Rat Anti-Mouse CD83 (1: 300) (eBioscience, San Diego, CA, USA). Limits for the quadrant markers were always set based on negative populations and isotype controls.

### Enzyme-Linked Immunosorbent Assay (ELISA)

Remnant colonic tissues (*n* = 8) were lysed in RIPA buffer (50 mM Tris-HCl at pH 7.4, 150 mM sodium chloride, 1% NP-40, 0.5% sodium deoxycholate, and 0.1% sodium dodecyl sulfate) with protease and phosphate inhibitor cocktail (Merck, Ashland, MA, USA) using a sonicator. Crude lysates were centrifuged at 20,000 × *g* for 20 min at 4°C. A part of supernatant (*n* = 8) was used to measure the level of GM-CSF, IL-4, IL-10, IL-12p70, IL-15, IL-23, IFN-γ, and TGF-β1 (eBioscience, San Diego, CA, USA) by commercial ELISA kits (eBioscience, San Diego, CA, USA) according to the manufacturer’s protocol. Each sample was tested in duplicate against the appropriate standard and optical densities measured by a microplate reader (BioRad, Hemel Hempstead, UK). Absorbance was read at 450 nm.

### Western Blot Analysis

Protein concentrations (*n* = 6) were determined in the supernatant of colonic tissues by classic BCA protein assay (Beyotime). Equal protein of each sample was fractionated onto sodium dodecyl sulfate polyacrylamide gel electrophoresis (SDS-PAGE) and transferred onto polyvinylidene fluoride (PVDF) membrane by a Bio-Rad Western blot apparatus. The membranes were blocked with 5% fat-free milk or 5% bovine serum albumin, and then probed with the following primary antibodies for 24 h at 4°C: GAPDH (1:2000), Anti-SOCS1(1:1000), Anti-SOCS3 (1:1000), Anti-JAK2 (1:1000), Anti-JAK2 (phospho Y1007 + Y1008) (1:500), Anti-STAT3 (1:1000), Anti-STAT3 (phospho Y705) (1:800), Anti-STAT6 (1:1000), Anti-STAT6 (phospho Y641) (1:800), Anti-PIAS3(1:1000) (Abcam, Cambridge, UK). The membranes were incubated with appropriate horseradish peroxidase-conjugated secondary antibodies (1:2000∼1:3000, Abcam, Cambridge, UK), and visualized with an enhanced chemiluminescence (ECL) detection kit (Millipore). Bands were quantified using Image-Pro Plus 5.0 software (Media Cybernetic, Bethesda, MD, USA).

### Statistical Analysis

Statistical analysis was performed using Prism 4.0 (Graph Pad Software, La Jolla, CA, USA). The data are expressed as mean ± standard error of mean (SEM). Student *t*-test or one-way analysis of variance (ANOVA) followed by the Tukey test for multiple comparisons. *P* values < 0.05 were considered statistically significant.

## Results

### Curcumin Attenuated TNBS-Induced Colitis

**FIGURE 1:**
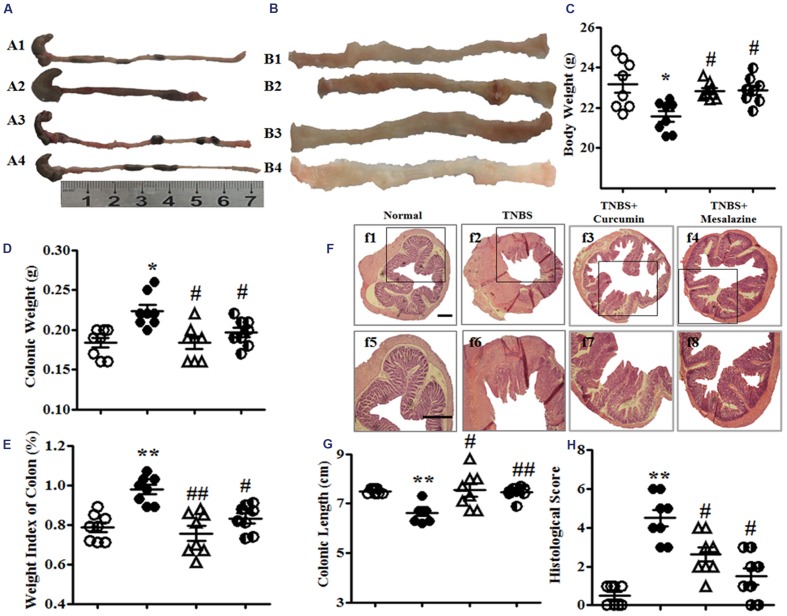
**Macroscopic and microcosmic observation.** **(A)** Typical images of an intact colon. A1, A2, A3, and A4, respectively, represent the Normal, 2, 4, 6-trinitrobenzene sulfonic acid (TNBS), TNBS + Curcumin, and TNBS + Mesalazine animal groups. **(B)** Macrography of the opened colon. B1, B2, B3, and B4, respectively, represent the Normal, TNBS, TNBS + Curcumin, and TNBS + Mesalazine animal groups. **(C)** Body weight. **(D)** Colonic weight. **(E)** Weight index of the colon. **(F)** Typical histological images stained by HE, f1–4: Bar = 40 μm, f5–8: Bar = 100 μm. **(G)** Colonic length. **(H)** Histological scores. Data were presented as mean ± SEM (*n* = 10). ^∗^*p* < 0.05 and ^∗∗^*p* < 0.01 versus the Normal group; ^#^*p* < 0.05 and ^##^*p* < 0.01 versus the TNBS group.

Administration of TNBS led to a severe illness characterized by loss of body weight, hematochezia, and diarrhea until the eight day. As shown in **Figure 1C**, the body weight of mice in the TNBS group was significantly decreased compared with the Normal group. However, the body weights of the mice with colitis treated with 100 mg/kg curcumin and 300 mg/kg mesalazine were markedly higher than the TNBS group.

Colonic weight and the weight index of the colon from the TNBS groups were higher than those in the Normal group, but they were lower than the TNBS + Curcumin and TNBS + Mesalazine groups (**Figures 1D,E**). However, the colonic length of colitis mice was shortened in the TNBS group compared with the Normal, TNBS + Curcumin, and TNBS + Mesalazine groups (**Figures 1A,G**).

Histological evaluation of colonic sections from untreated animals with colitis showed that TNBS-induced colitis was characterized by a loss of mucosal architecture, thickening of the colon wall, cryptic abscesses, the formation of ulcers, and extensive inflammatory cell infiltration in the colonic mucosa (**Figure 1F**). Treatment with curcumin and mesalazine restrained these pathological symptoms and histo-progressive restoration, reduced inflammatory cell infiltration in the mucosa and submucosa, and maintained the integrity of colonic mucosa (**Figure 1F**). While ulceration, hyperaemia, and edema in local colonic mucosa in colitis mice without treatment were observed by visual assessment, they were ameliorated in colitis mice treated with curcumin and mesalazine (**Figure 1B**). Moreover, the histological scores in the colon of mice from the Normal, TNBS + Curcumin, and TNBS + Mesalazine groups were significantly lower than those in untreated mice with colitis (**Figures 1F,H**). All results demonstrated that curcumin effectively treated experimental colitis.

### Curcumin Decreased the Total Number of DCs in PPs

**FIGURE 2:**
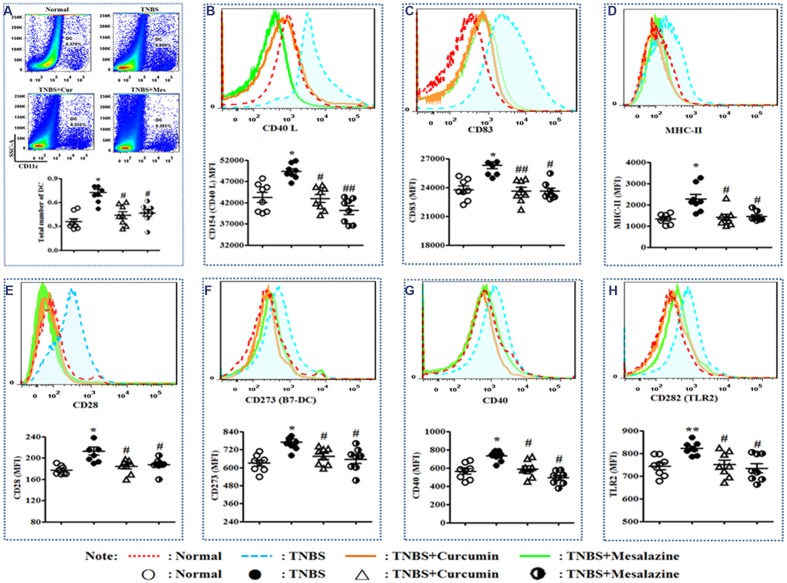
**Typical pseudocolor and level of costimulatory molecules of Dendritic cell (DC).** **(A)** Typical pseudocolor and totality (%) of DC. **(B)** Typical pseudocolor and level (MFI) of CD40 L. **(C)** Typical pseudocolor and level (MFI) of CD83. **(D)** Typical pseudocolor and level (MFI) of MHC-II. **(E)** Typical pseudocolor and level (MFI) of CD28. **(F)** Typical pseudocolor and level (MFI) of CD273 (B7-DC). **(G)** Typical pseudocolor and level (MFI) of CD40. **(H)** Typical pseudocolor and level (MFI) of CD 282 (TLR2). Data were presented as mean ± SEM (*n* = 8). ^∗^*p* < 0.05 and ^∗∗^*p* < 0.01 versus the Normal group; ^#^*p* < 0.05 and ^##^*p* < 0.01 versus the TNBS group.

In **Figure 2A**, the total number of DCs in the PPs of colitis mice without treatment was higher than that of normal mice and colitis mice treated with curcumin and mesalazine.

### Curcumin Regulated Costimulatory Molecules in PPs

Costimulatory molecules are specific markers of mature DCs. The main costimulatory molecules of DCs were analyzed by FCM. Elevated expressions of CD40L (**Figure 2B**), CD83 (**Figure 2C**), MHC-II (**Figure 2D**), CD28 (**Figure 2E**), CD273 (B7-DC) (**Figure 2F**), CD40 (**Figure 2G**), and CD282 (TLR2) (**Figure 2H**) were demonstrated in the TNBS group compared to the Normal, TNBS + Curcumin, and TNBS + Mesalazine groups (**Figure 2**).

### Curcumin Regulated Cytokine Expression in Colonic Tissues

**FIGURE 3:**
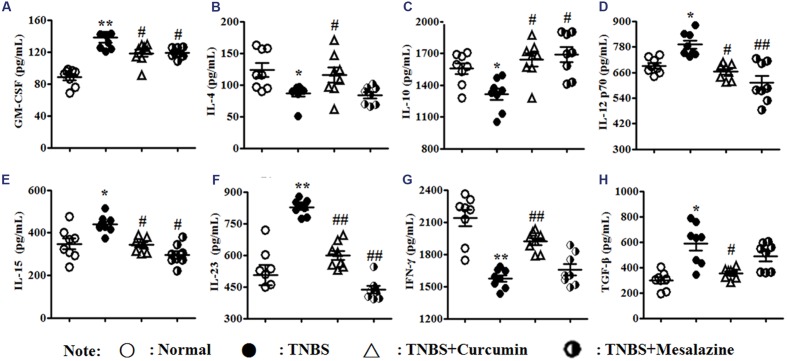
**Concentrations of cytokines in colonic mucosa.** Concentrations of cytokines **(A)**, Granulocyte macrophage colony-stimulating factor (GM-CSF). **(B)**, Interleukin (IL)-4. **(C)**, IL-10. **(D)**, IL-12 p70. **(E)**, IL-15. **(F)**, IL-23. **(G)**, IFN-γ. **(H)**, TGF-β1 in colonic mucosa from different groups. Data are presented as mean ± SEM (*n* = 8). ^∗^*p* < 0.05 and ^∗∗^*p* < 0.01 versus the Normal group; ^#^*p* < 0.05 and ^##^*p* < 0.01 versus the TNBS group.

In the development of DCs, the expression of cytokines is indispensable to the microenvironment of maturation. The levels of GM-CSF (**Figure 3A**), IL-12p70 (**Figure 3D**), IL-15 (**Figure 3E**), IL-23 (**Figure 3F**), and TGF-β (**Figure 3H**) in untreated mice with colitis were elevated compared to those of the Normal, TNBS + Curcumin, and TNBS + Mesalazine groups. However, expressions of IL-4 (**Figure 3B**), IL-10 (**Figure 3C**), and IFN-γ (**Figure 3G**) in the colonic mucosa of the TNBS group were attenuated compared to the Normal, TNBS + Curcumin, and TNBS + Mesalazine groups.

### Effect of Curcumin on the Signaling Pathway of JAK/STAT/SOCS in Colonic Tissues

**FIGURE 4:**
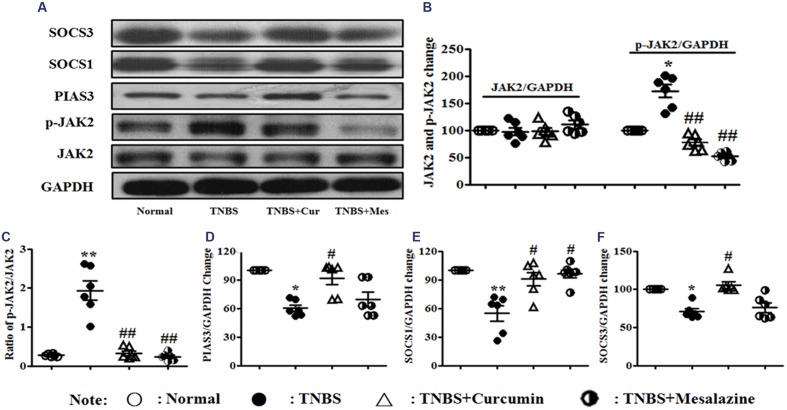
**Western blot analysis of Janus kinase (JAK)2, p-JAK2, PIAS3, suppressors of cytokine signaling (SOCS)1, and SOCS3** **(A)** Western blot of JAK2, p-JAK2, PIAS3, SOCS1, and SOCS3. **(B)** Quantitative analysis of JAK2 and p-JAK2. **(C)** Ratio of p-JAK2/JAK2. **(D)** Quantitative analysis of the PIAS3 protein. **(E)** Quantitative analysis of the SOCS1 protein. **(F)** Quantitative analysis of the SOCS3 protein. Data are presented as mean ± SEM (*n* = 6). ^∗^*p* < 0.05 and ^∗∗^*p* < 0.01 versus the Normal group; ^#^*p* < 0.05 and ^##^*p* < 0.01 versus the TNBS group.

The JAK/STAT/SOCS signaling pathway diffusely participates in differentiation, maturation, and activation of DCs. The expression of related proteins was measured by Western blot. It can be shown in **Figure 4** that the activation of phospho-JAK2 (**Figures 4A–C**) was inhibited, and the expressions of PIAS3 (**Figures 4A,D**), SOCS1 (**Figures 4A,E**), and SOCS3 (**Figures 4A,F**) were increased in the colonic mucosa from colitis mice treated by curcumin. Meanwhile, the ratio of p-JAK2/JAK2 (**Figures 4A–C**) was decreased after mice with experimental colitis were treated for 7 days.

**FIGURE 5:**
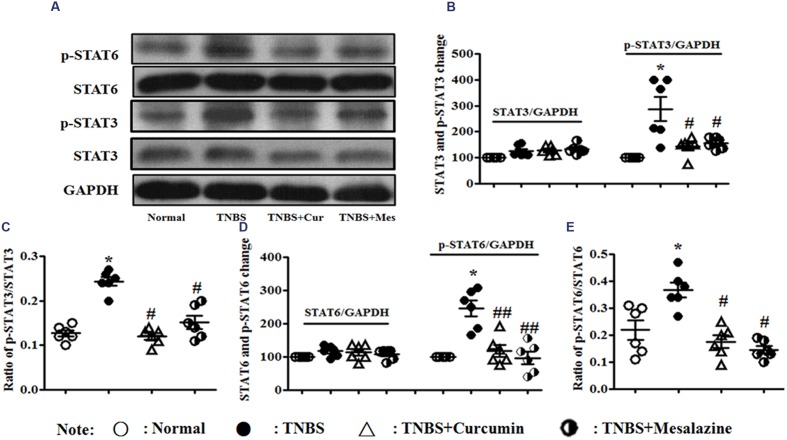
**Western blot analysis of signal transducer and activator of transcription (STAT)3, p-STAT3, STAT6, and p-STAT6.** **(A)** Western blot of STAT3, p-STAT3, STAT6, and p-STAT6 protein. **(B)** Quantitative analysis of STAT3 and p-STAT3 protein. **(C)** Ratio of STAT3/p-STAT3. **(D)** Quantitative analysis of STAT6 and p-STAT6 protein. **(E)** Ratio of STAT6/p-STAT6. Data are presented as mean ± SEM (*n* = 6). ^∗^*p* < 0.05 versus the Normal group; ^#^*p* < 0.05 and ^##^*p* < 0.01 versus the TNBS group.

However, in **Figure 5**, the levels of phospho-STAT3 (**Figures 5A–C**) and phospho-STAT6 (**Figures 5A,D,E**) were up-regulated in the colonic mucosa of mice from the TNBS, which was in contrast to the Normal, TNBS + Curcumin, and TNBS + Mesalazine groups. The ratios of phospho-STAT3/STAT3 (**Figures 5A–C**) and phospho-STAT6/phospho-STAT6 (**Figures 5A,D,E**) mirrored the levels of phospho-STAT3 and phospho-STAT6. In other words, because colitis was treated with curcumin for 7 days, these ratios were down-regulated as compared to colitis mice without treatment.

## Discussion

Colitis induced by TNBS in mice is a widely used and accepted experimental model to explore novel pharmacological approaches for preventing and treating colitis in humans (Harusato et al., 2013; Sun et al., 2015). Key characteristics of chronic TNBS colitis include a predominant Th1-mediated immune response with loss of mucosal architecture, crypt abscesses, infiltrations of lymphocytes/macrophages, and thickening of the colonic wall (Wirtz et al., 2007). As initiators of acquired immunity and regulators of self-tolerance, intestinal DCs widely distributed throughout the LP and PP (Steinman and Nussenzweig, 2002). After TNBS treatment, increased numbers of DCs were recruited to the inflammatory sites in the gut from days 1–3, and lower expression of costimulatory molecules occurred along with the termination of TNBS-induced inflammation (Hoshino et al., 2010). These data suggest that the termination of inflammatory injuries was potentially controlled by DCs. In the present study, curcumin treatment alleviated pathological damage and colon weight reduction, extended the length of the colon, and decreased the weight index of the colon and the histological score in TNBS-induced colitis. In addition, we determined that the number of DCs in the PPs of colitis mice without treatment was higher than that of Normal mice or mice treated by curcumin. These results hinted that it was a positive correlation between the therapeutic effects of curcumin treated colitis and down-regulated levels of DC.

As the most powerful antigen-presenting cells, DCs are critical for the regulation of intestinal immunity and mucosal immune tolerance to commensal microorganism, which are one of the pivotal inflammatory etiologies induced by UC and CD (Stagg et al., 2003). Over-expression of MHC-II and costimulatory molecules from DCs, and maturation or migration of DCs in peripheral lymph nodes are “danger” signals that induce inflammatory mucosal injuries in the gut (Grewal and Flavell, 1998; Theill et al., 2002). Mature DCs secrete costimulatory molecules that can regulate the balance between Th1 and Th2. The abnormal generation of polarized Th1 and Th2 responses induces inflammatory damage in the gut as IBD. Pathways of Th1 and Th2 response are regulated by DCs, which include costimulatory molecules and related cytokines (Stagg et al., 2003). Costimulatory molecules of DCs are composed of TNF/TNF receptor protein families (CD40/CD40L, OX40/OX40L, and TNFR/TNF) and the immune globulin superfamily (ICAM-1/LAF-1, CD28/CTLA4/B7, etc.), which supply secondary signaling in the activation and polarization of T cells (Grewal and Flavell, 1998; Theill et al., 2002). CD40/CD40L signaling can promote DCs to secrete IL 12 (IL-12) to further differentiate CD4^+^T cells to Th1. A similar function was shown in the ICAM-1/LFA-1 signal and B7-1 molecule (B7/CD28 signal). However, the B7-2 molecule and OX40/OX40L signal promotes polarization of Th2 cells. These costimulatory molecules are highly expressed in human and animal colitis (Griseri et al., 2010; Vainer, 2010).

Inflammatory bowel disease was induced by the over-expression of pro-inflammatory cytokines (TNF-α, IL-1, IL-6, and IL-12) and/or the down-regulation of anti-inflammatory cytokines in the polarization of Th1/Th2 cells in colonic mucosa (Kallies et al., 2006; Martins et al., 2006). Their changes of these cytokines secreted by DC and other cells, are probable results of DCs activation and maturation, or/and effect on this process of DC. In the present study, the level of MHC-II was increased in DCs from the colitis mice without treatment along with over-expression of costimulatory molecules of DCs, including CD83, CD28, B7-DC, CD40, CD40 L, and TLR2. These results indicated that DCs in mice were matured or activated in TNBS-induced colitis. In the TNBS group, the levels of Th2 cytokines (IL-4, IL-10) were decreased and the secretion of IL-12 p70 was increased. Mature DCs expressed costimulatory molecules and were stimulated by these molecules to polarize T cells, leading to an imbalance of Th1/Th2 and inflammatory colonic damage to form ulcers and colitis. After treatment with curcumin for 7 days, these DC costimulatory molecules (CD83, CD28, B7-DC, CD40, and TLR2) were inhibited, and followed with the up-regulated levels of IL-4 and IL-10, and the down-regulated levels of IL-12 p70. The present research certified that curcumin reduced the up-regulation of costimulatory molecules and restrained the activation of DCs. Furthermore, curcumin inhibited pro-inflammatory cytokines and promoted DCs to rectify Th1 and Th2 balance in the therapeutic process of TNBS-induced colitis in mice.

The function of DCs has the potential to be modulated through genetic engineering affecting the JAK/STAT signaling pathway (Säemann et al., 2003). The JAK/STAT signaling pathway is involved in immune function and cell growth or differentiation. The four members of the Janus family kinases (Jaks), JAK1, JAK2, JAK3, and TYK2, are expressed ubiquitously in mammals, and JAK3 is primarily expressed in hematopoietic cells (Lu et al., 2008; Xiong et al., 2008). JAKs are critically involved in the growth, survival, development, and differentiation of immune cells, including T cells, B cells, and DCs. Once binding of a cytokine to its cognate receptor triggers tyrosine phosphorylation and activation of the JAKs, JAKs serve as docking sites for signaling molecules such as STATs, including STAT 1, 2, 3, 4, 5A, 5B, and 6. JAKs phosphorylate STATs. Activated STATs have essential roles in transmitting many cytokine-mediated signals and thereby have similar crucial roles in Th cell differentiation (Zhu et al., 2010). However, the JAK/STAT signaling pathway is activated when cytokines are bound together with their receptors and inhibited by SOCS, which interrupt the process of cytokine and receptor binding (Slattery et al., 2014). Thus, SOCS proteins have been defined as an important mechanism for the negative feedback system of the JAK/STAT pathway (Starr et al., 1997; Kubo et al., 2003). The JAK/STAT/SOCS-signaling pathway, which is an important regulator of the ultimate cellular response to cytokines, can interact with cytokines, just as pro-inflammatory cytokines (such as TNF-α, IL-6, and INF-γ) can up-regulate STAT protein (Pfitzner et al., 2004; Shea-Donohue et al., 2010). These responses are closely associated with inflammatory processes in colonic mucosa and lead to colitis (Wang et al., 2009; Tetreault et al., 2012). The prevention of tissue damage is essential to control the magnitude and duration of JAK/STAT signaling (Shuai and Liu, 2003).

Research has indicated that the JAK/STAT signaling pathway modulates the differentiation of the T cells by regulating the maturation, function, activation, and migration of DCs. Accompanied by the increased expression of costimulatory molecules (CD80, CD83, and CD86) and MHC-II (Guermonprez et al., 2002), DCs mature when stimulated by granulocyte macrophage colony-stimulating factor (GM-CSF) under induction of TLR (Strengell et al., 2006). In this process, the IL-12 receptor binds to JAK proteins activate STAT1, STAT3, and STAT4. The downstream signaling of IL-12 inversely stimulates expression of SOCS1 and SOCS3 (Bartz et al., 2006). Normally, over-expressed SOCS1 and SOCS3 in the TLR pathway can inhibit GM-CSF signaling and suppress DC maturation. SOCS1 inhibits the JAK2 phosphorylation, and restrains DC maturation which can induce GM-CSF through JAK signaling (Bartz et al., 2006; Strengell et al., 2006). Zhong et al. (2010) recently suggested that JAK2 selectively regulates the capacity of DCs to initiate immune responses. Other studies have demonstrated that IFN-γ-induced JAK1 activation in human APCs enhances the IL-12p70 level and inhibits IL-10 release, revealing a potential target of immunosuppressive strategies (Conzelmann et al., 2010).

Silencing of SOCS1 enhances antigen presentation of DCs. SOCS1-silenced DCs produce enhanced levels of IL-12p70 and induce a hyper–Th1-type immune response, compromising self-tolerance to cause pathological autoimmune responses (Evel-Kabler et al., 2006; Sabado and Bhardwaj, 2010). SOCS1 is an essential and negative control protein of DC-mediated T cell activation and continuous immune regulation. Moreover, increased expression of SOCS3 in DCs can reduce expression of STAT3 and decrease the production of IL-12 and IFN-γ, inducing a Th2 cell response (Li et al., 2006).

Dendritic cells are likely to be pivotal in the balance between tolerance and active immunity to commensal microorganisms that are fundamental pathogens in IBD (Stagg et al., 2003). DCs up-regulate their expression of MHC-II and costimulatory molecules and undergo maturation and migrate to peripheral lymph nodes, such as PPs. These mature DCs can produce IL-12, IL-18, and IL-23 to polarize Th1 and Th2 responses. This phenomenon is local and results in dangerous information in the colon that causes inflammatory damage and ulcer formation (Stagg et al., 2003). The JAK/STAT pathway has been associated with cell migration and also affects chemokine production. Specifically, different JAK proteins become tyrosine-phosphorylated upon stimulation with various chemokines in different cell types (Stagg et al., 2003). Recent studies suggest that JAKs, including JAK1, JAK2, and JAK3, are involved in proper DC migration to secondary lymphoid organs (Rivas-Caicedo et al., 2009; Yarilina et al., 2012). Recently, JAK/STAT/SOCS signaling pathway has been identified as a multi-target to influence DC function, maturation, and migration.

In the present study, 7 days after TNBS-induced colitis was treated by curcumin, colonic mucosa damage was attenuated, phosphorylation of the three members (JAK2, STAT3, and STAT6) in the JAK/STAT/SOCS signaling pathway were inhibited, and the three downstream proteins (SOCS1, SOCS, and PIAS3) of this signaling pathway were highly expressed. Simultaneously, the levels of GM-CSF, IL-12p70, IL-15, and IL-23 were decreased, and up-regulation of IL-4, IL-10, and IFN-γ occurred after 7 days of treatment with curcumin. We found that curcumin inhibited phosphorylation of JAK2, STAT3, and STAT6 in low-levels of GM-CSF and TLR2, and increased expressions of PIAS3, SOCS1, and SOCS3 to suppress activation of the JAK/STAT signal. Markers of DC maturation, MHC-II and costimulatory molecules of DCs were down-regulated. Our results indicated that inactivation of the JAK/STAT signal limited the extent of DC maturation and function, and reduced the quantity of DCs. For curcumin treatment, the function of DCs, including the secretion of cytokines and antigen presentation, was limited, and then led to a decrease in the expression of pro-inflammatory cytokines (IL-12 p70, IL-15, and IL-23), and to an increase in the level of IL-4, IL-10, and IFN-γ. This process leads to rectified abnormal polarization of Th1 and Th2 responses and alleviated inflammatory colonic damage to effectively treat experimental colitis.

However, many documents had shown that increased levels of IFN-γ were found in TNBS-induced colitis. IFN-γ is not a sole factor resulted in TNBS-induced colitis. Some researcher found that the severity of colitis was higher in IFN-γ KO mice induced by TNBS, and deduced that IFN-γ was not an important mediator of the local inflammation response (Jin et al., 2012). IFN-γ is double-acting in the different phase of the pathogenetic process of colitis. Sheikh et al. (2010) had demonstrated that IFN-γ has anti-inflammatory properties in the initiation phase of IL-23 – mediated experimental colitis. The results hinted that IFN-γ had protective effects on the experimental colitis (Sheikh et al., 2010). In the present study, we found that curcumin increased levels of IFN-γ in mice colitis, which is possibly correlated with different time phase of experimental colitis. While TGF-β1 expression was inhibited by curcumin in the present study, TGF-β is a multifunctional set of peptides that controls proliferation, differentiation, and other functions in many cell types. It is known that TGF-β can inhibit immunocompetent cell proliferation and lymphocyte differentiation, and promote fibroblast cell proliferation to induce chronicity and fibrosis of disease. These results hinted that curcumin potentially prevent from the chronicity of colitis. Certainly, the results should be verified repeatedly and explored their pathway in colitis treatment in the next work.

## Conclusion

Curcumin suppressed the activation of DCs by modulating the JAK/STAT/SOCS signaling pathway to restore immunologic balance and to treat experimental colitis in an effective manner.

## Author Contributions

Conceived and designed the experiments: D-YL and H-MZ. Performed the experiments: H-MZ, RX, X-YH, S-MC, M-FH, H-YY, XW, and YZ. Contributed reagents/materials/analysis tools: D-YL and H-MZ. Analyzed the data: D-YL and A-PL. Wrote the paper: D-YL and H-MZ. All authors contributed to and approved the final draft of the manuscript.

## Conflict of Interest Statement

The authors declare that the research was conducted in the absence of any commercial or financial relationships that could be construed as a potential conflict of interest.

The reviewer EK and handling Editor declared their shared affiliation, and the handling Editor states that the process nevertheless met the standards of a fair and objective review.
